# Preparation of plasticized poly (lactic acid) and its influence on the properties of composite materials

**DOI:** 10.1371/journal.pone.0193520

**Published:** 2018-03-01

**Authors:** Decai Li, Yang Jiang, Shanshan Lv, Xiaojing Liu, Jiyou Gu, Qifeng Chen, Yanhua Zhang

**Affiliations:** College of Material Science and Engineering, Northeast Forestry University, Harbin, PR China; Institute of Materials Science, GERMANY

## Abstract

Plasticized poly (lactic acid) (PPLA) was prepared by melt blending poly (lactic acid) (PLA) with 10 wt% of poly (ethylene glycol) (PEG), with varied molecular weights range from 400 to 4000. The structure, thermal property, morphology, and surface free energy of the PPLA were investigated by Fourier transform infrared spectroscopy (FTIR), differential scanning calorimeter (DSC), thermogravimetric analysis (TGA), scanning electron microscopy (SEM), and contact angles (CA). The resulting PPLA results indicated that the introduction of PEG to the blend systems resulted in a ductile fracture, a decrease in the melt temperature (*T*_m_) and glass transfer temperature (*T*_g_), and an increase in the degree of crystallization (*χ*_c_), which indicated an improved flexibility. In addition, the polarity of the PPLA increased and the surface free energy decreased. The resulting PPLA was subsequently used as matrix to blend with wood flour to prepare composites. The mechanical strength, melting behavior, thermal stability, and microscopy of the PPLA/wood flour composites were also evaluated. These results illustrated that the plasticized PPLA matrix was beneficial to the interfacial compatibility between the polar filler and the substrate.

## Introduction

In the past few decades, irreparable damages to the environment caused by petroleum-based polymers have led to increased attentions on the research and development of renewable and biodegradable polymer composites [1]. Demands of biodegradation and reproducibility have driven series studies on thermoplastic polymers such as poly (lactic acid) (PLA) to produce fiber-PLA materials using injection molding [2–4]. To reduce the cost of the biopolymers, efforts have been made to blend PLA with selected natural fibers[5]. Among them, wood flour (WF), which is derived from natural resources, has many advantages such as various forms, large quantities, light, cheap, and renewable, and can be added to various matrices in considerable amounts, thereby providing economically favorable solutions [6–9]. The main problem of such composites is the poor adhesion to all major polymer matrices.

Due to the poor compatibility between the hydrophilic fiber and the hydrophobic PLA matrix, numerous of formulations based on plasticizers were explored as the most simple and effective way. Various types of compounds have been reported as plasticizers for PLA. Among the various plasticizers, polyethylene glycol (PEG) is one of the efficient plasticizers used for polymers because of the advantage of a broad range of molecular weights, nontoxic, miscibility, and biodegradability [10–13]. The plasticization of PEG can effectively increase the chain mobility of PLA, and improve its ductility and drawability, thus broadening the range of potential applications. It should be pointed out that the plasticizing modification of PEG is mainly aimed at the property modification of the polymers and the expansion of the application fields of PLA. Although considerable amounts of studies have been conducted on PLA plasticized with PEG, comprehensive studies on its composite materials of WF/PLA prepared by a plasticizing system with PEG have been rarely reported.

The main aim of this study was to improve a plasticizing method for PLA matrix and thereby to propose new insights into the preparation of fiber-PLA composites for compatibility modification. In this study, PLA resin was blended with PEG with different molecular weights (*M*_w_), and the tensile properties, morphologies, crystallization behaviors, and surface free energy of the resulting PPLA were investigated. In order to evaluate the plasticization effect of PEG on the final products of WF/PPLA composites, the compatibility, mechanical strength, thermal and thermal stability of the composites were studied at the same time. This paper should provide an efficient method to design the PLA-based composites and improve the possibility to enhance the processability and expand the application of PLA materials.

## Experimental

### Materials and blend preparation

Poly (L-lactic acid) (grade 306D) with a melt index of 16 g/10 min^-1^ (160°C, 2.16 kg) and a density of 1.2 ± 0.05 g·cm^-3^ was obtained from Ningbo Huanqiu Plastic Products Co., Ltd. (Ningbo, Zhejiang, China). The average molecular weight was approximately 1.8 × 10^6^–2.0 × 10^6^. Four PEGs with a nominal *M*_w_ equal to 400 g·mol^-1^ (PEG 400) to 1000 g·mol^-1^ (PEG 1000) and 2000 g·mol^-1^ (PEG 2000) to 4000 g·mol^-1^ (PEG 4000) were used as plasticizers. The pieces of wood were pulverized through a pulverizer to the size of 80–100 mesh wood flour which was supplied by Baiquan Wood plastic composite material base (HeilongJiang, China).

Prior to blending, plasticizers were dissolved in ethanol to allow full mixing with the PLA matrix; the ethanol solvent was completely volatilized during the placed process. The PEG/PLA blends (PPLA) were made by extrusion molding in which the raw materials were first passed through a co-rotating twin-screw extruder(L/D ratio of 40,Nanjing Giant SHJ-20), while the temperatures were set at 135-150-170-170-135°C from the feeder zone to the die. The granules were then fed into an injection molding machine to obtain the dumbbell-shaped samples, while the temperatures of the four sections were set at 180-180-170-170°C.

The preparation process of the PPLA/WF composites was similar to that of the plasticized PPLA blends. Prior to that, various amounts of PEG plasticizer(between 1 and 9% based on the total weight of the PLA and PEG), were mixed together with the PLA particles by using a twin-screw extruder. The plasticized PLA was evaluated as a matrix resin and then blended with WF at the ratio of 3:7, and then followed by extrusion granulation and injection molding. The temperature of the former was unchanged, while the injection molding temperature was set at 165-175-170-160°C. All specimens were maintained at room temperature for one week.

### Characterization

#### Fourier transform infrared spectrometer (FTIR)

The analysis of samples was carried out in the 400–4000 cm^-1^ range in attenuated total reflection (ATR) mode with a Magna-IR 560 E.S.P. (Thermo Nicolet Co., USA). The results were obtained by averaging 32 scans at a resolution of 4 cm^-1^. Prior to each test, a background spectrum was obtained to compensate for the humidity effect and the presence of carbon dioxide by spectra subtraction.

#### Differential scanning calorimeter (DSC)

The DSC 214 instrument differential scanning calorimeter (NETZSCH Co., Germany) was used to evaluate the modifications of the chemical structure of the plasticized PLA. The scanning temperature ranged from 25 to 200°C at a rate of 10°C·min^-1^ under a nitrogen atmosphere (flow rate 20 ml·min^-1^). After the first heating period in the range of 25 to 200°C, the samples were cooled to 25°C and subsequently heated again at the same rate. The crystallinity was calculated according to Eq (1) [14–16]

$$\chi_{c}=\frac{\Delta Hm}{\Delta H_{m}^{0}\times \varphi_{PLA}}\times 100\%$$ (1)

where *χ*_c_ is the crystallinity of the PLA in the blend, Δ*H*_m_ is the fusion heat (J·g^-1^), ΔH_m_^0^ is the fusion heat of 100% crystallinity of the PLA, taken as 93.6 J·g^-1^, and Φ_PLA_ is the weight fraction of the PLA in the blend (90% in this study).

#### Thermogravimetric analysis (TGA)

The TGA measurements were carried out with a TG 209 F1 (NETZSCH Co., Germany) to study the thermal stability. Specimens (about 5 mg) were heated from room temperature to 600°C with a heating rate of 10°C/min under argon gas flow.

#### Mechanical properties

The properties of the blends were tested on a CMT-5504 Universal Testing Machine (Shenzhen SANS Test Machine, China), according to the GB/T 1040–2006 and GB/T9341-2000 (China) method respectively. The tensile and flexural properties of the blends were tested at a crosshead speed of 10 mm/min.

#### Contact angles (CA)

The contact angles (CA) were measured by the sessile drop technique using a contact angle system (OCA20, Dataphysics, Germany) at room temperature. Surface free energy values as the sum of dispersion and polar components were calculated from the contact angles using the geometrical mean method according to the equation derived by Owens-Wendt [17, 18], as shown in Eq (2). The testing liquids were water, ethylene glycol, and atolin and the parameters are shown in Table 1.

$$\gamma_{L}\left(1+COS\theta \right)/\left(2\cdot \sqrt{\gamma_{L}^{D}}\right)=\sqrt{\gamma_{S}^{P}}\cdot \sqrt{\left(\gamma_{L}^{P}/\gamma_{L}^{D}\right)}+\sqrt{\gamma_{S}^{D}}$$ (2)

where L and S refer to the liquid and solid, respectively; *γ*^D^ is the dispersive component and *γ*^P^ is the polar component of the surface energy; *θ* is the contact angle. Eq (2) can be solved by using three liquids with known *γ*^D^ and *γ*^P^.

**Table 1:** Values of surface free energies for the test liquids.

Liquid	Surface free energy (mN/m)		
	γ_L_^D^	γ_L_^P^	γ_L_
Water	21.8	51.0	72.8
Ethylene glycol	29.3	19.0	48.3
Atolin	28.9	0	28.9

#### Scanning electron microscopy

A JSM-7500F SEM operating at an accelerating voltage of 20 kV was used to observe the micro-morphology of the fractured samples after tensile testing. The fracture surfaces were tested after being sputter-coated with gold.

## Results and discussion

### Characterization of PLA blends

**Fig 1:**
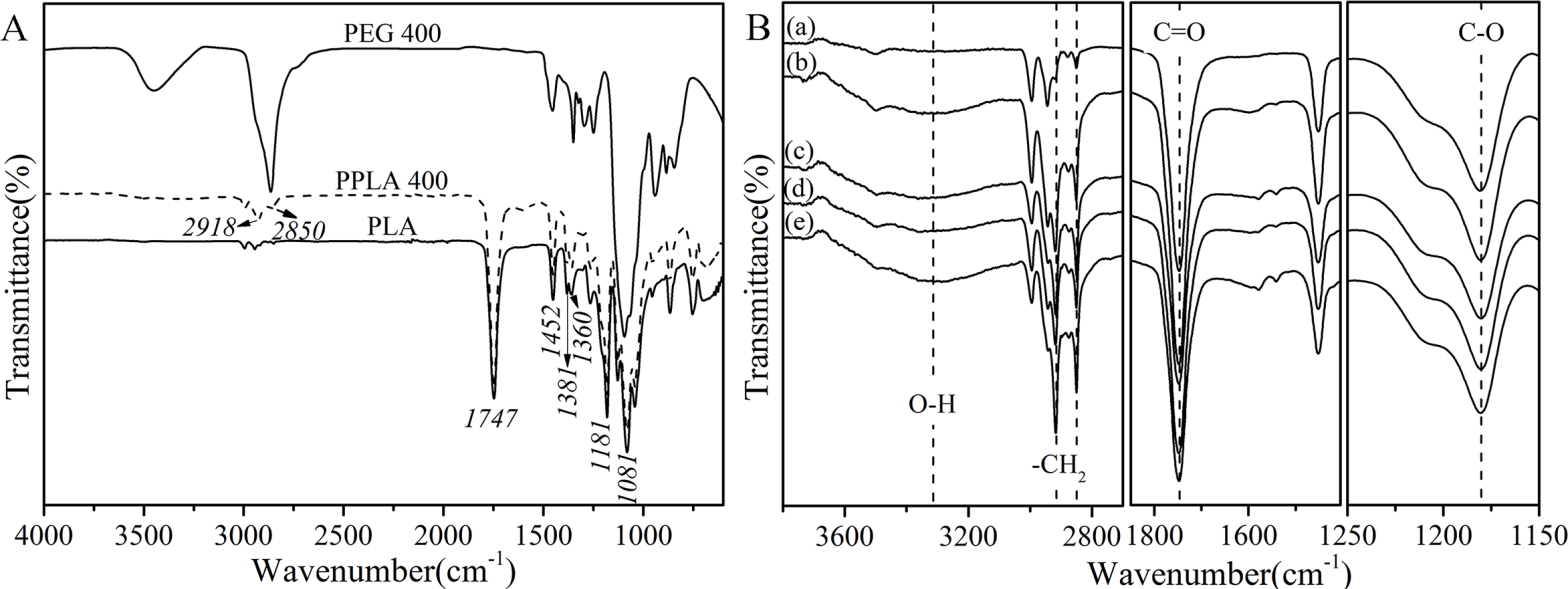
ATR-FTIR spectra of (A) the native PLA, PEG 400, PPLA 400 and (B) PPLA with different *M*_w_: (a) neat PLA, (b)PPLA 400, (c) PPLA 1000, (d) PPLA 2000, (e) PPLA 4000.

The FTIR spectra (Fig 1) depict the chemical structural changes in the PLA blends before and after plasticization. As shown in Fig 1A, both PEG 400 and PPLA 400 have two adjacent peaks located at 2918 and 2850 cm^-1^, which are assigned to absorption of -CH_2_-stretching vibrations. Additionally, the increase in the intensity at 1381 and 1360 cm^-1^ (representing the C-H deformation peak) indicated the existence of a -CH_2_ chain segment of the PEG. For PLA and PPLA 400, the characteristic absorption of the C = O stretch and the C-O stretch of the ester group appeared at 1747 and 1181 cm^-1^ respectively. To analyze the influence of the different *M*_w_ of the PEGs on the structural transformation of the PLA in more detail, the blends were characterized by IR (Fig 1B). As to the spectra of the plasticized blends, the intensity of the bands at 3318, 2918, and 2850 cm^-1^ increased, while the ratio of the C-O and C = O peak areas increased at the same time, especially for the PPLA 4000. This may suggest that part of the H-O groups of the PEG form hydrogen bonds with the C = O groups from the PLA. As a result, the C-O groups and hydrogen bonds increased, which is consistent with previous experimental results [19].

### Mechanical properties of PLA blends

**Fig 2:**
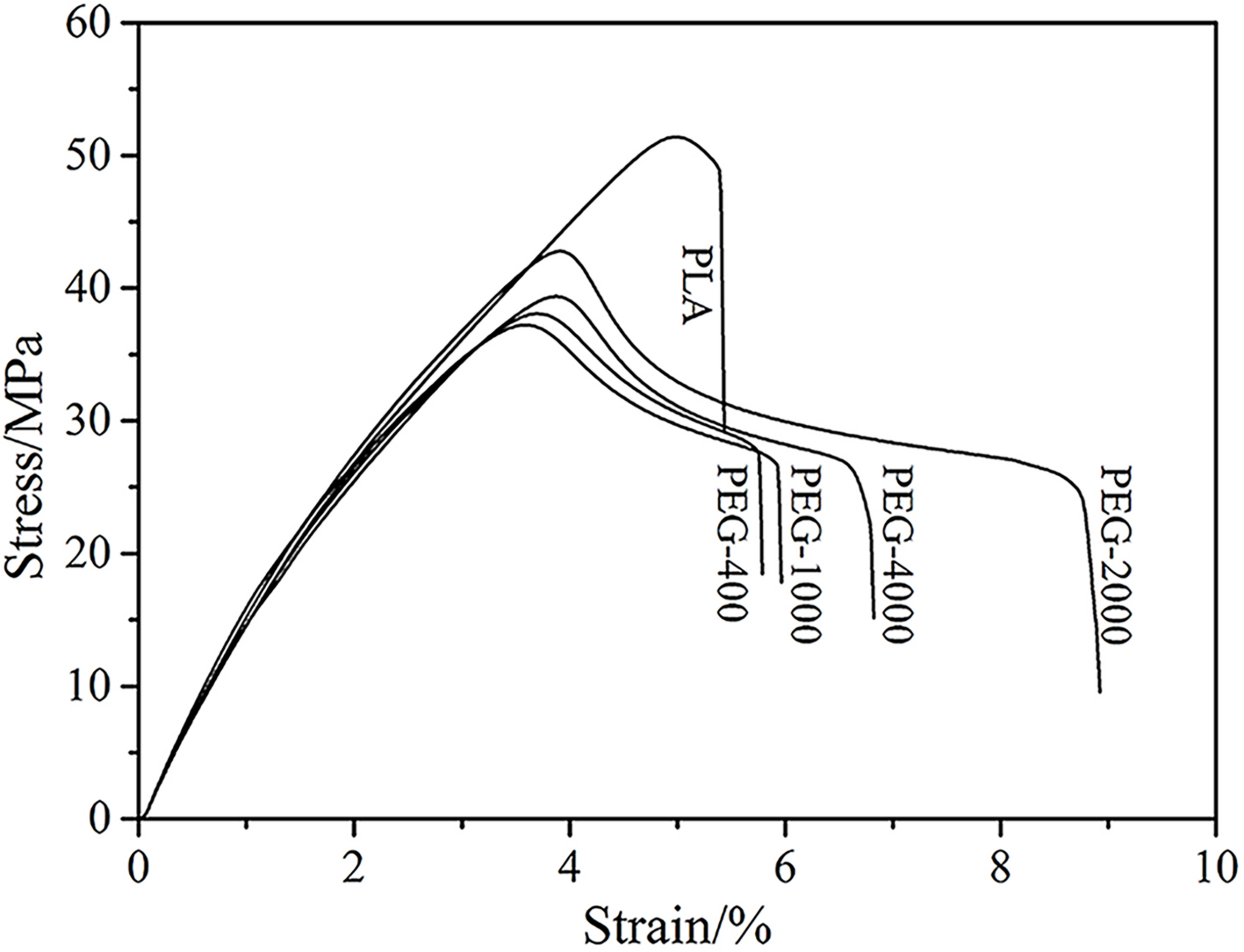
Tensile stress-strain curves of PLA plasticized by PEG with different molecular weight.

**Fig 3:**
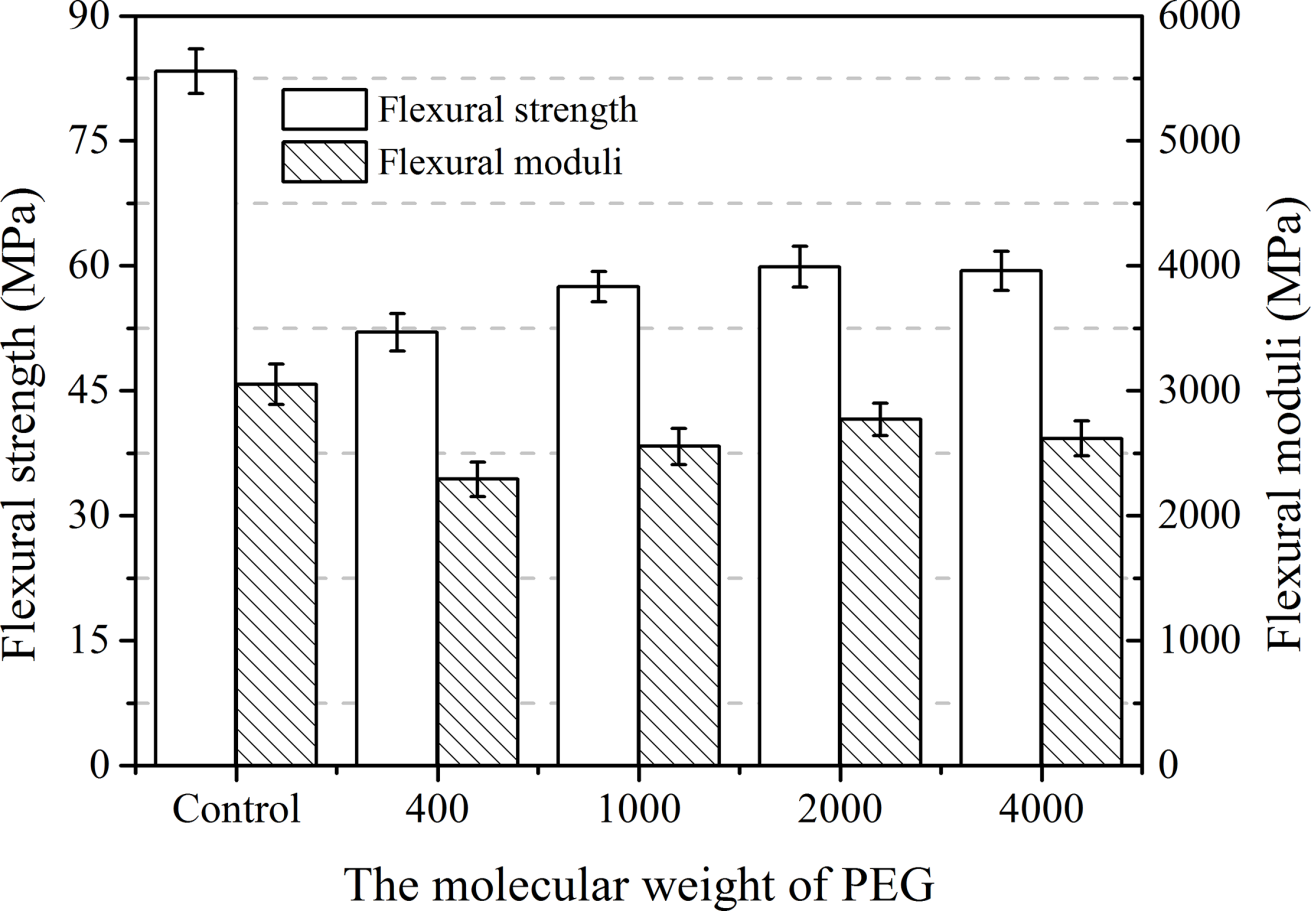
Flexural strength and moduli of PLA plasticized by PEG with different molecular weight.

The effect of adding PEG on the mechanical properties of the PLA blends was studied and the representative stress-strain curves and flexural properties of the blends are presented in Fig 2 and Fig 3 respectively. Regarding the blends, the addition of 10 wt% PEG with different *M*_w_s to the PLA resulted in a considerable increase in the elongation at break and a decrease in the tensile strength; these results were consistent with previous experimental results [20–22]. This occurred because, during the blending process with the PLA, the small molecular weight of the PEG was equivalent to the solvent, resulting in the increased free volume of the polymer and the decrease in the material’s stiffness [23]. When the *M*_w_ of the PEG was increased, a considerable increase in the elongation occurred while the tensile strength remained unchanged. This result indicated that the addition of 10 wt% PEG was necessary to obtain a good plasticizing effect, which may be due to the decrease in crystallinity analyzed below [24]. It is noteworthy that an addition of 10 wt% PEG 2000 to the PLA facilitated the plasticizing effect.

Fig 3 displays the relationship between the flexural properties and the PEG’s *M*_w_. Clearly, both flexural strength and flexural modulus decreased as expected due to the presence of the PEG with different *M*_w_s. However, a slight but significant increase was noted as the M_w_ of PEG increased to 2000. Beyond this value, however, the strength and modulus began to decrease slightly. The results were similar to that of the tensile test. In summary, the results clearly indicate that the addition of PEG has a plasticizing effect on the PLA matrix, and the plasticized PLA blend exhibited optimum properties with PEG 2000 as the plasticizer.

### PPLA morphologies

**Fig 4:**
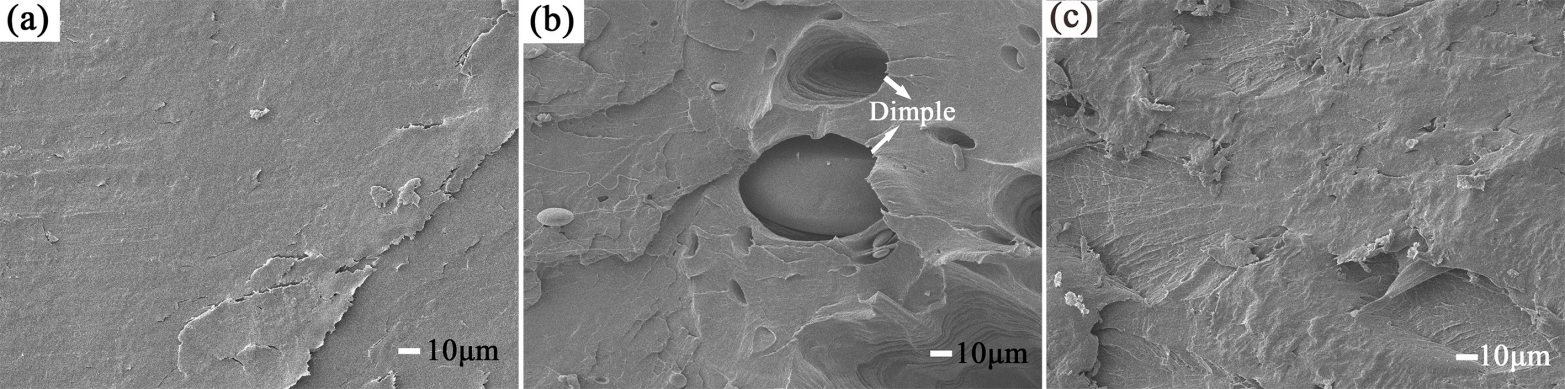
SEM micrographs of tensile fracture surface: (a) PLA, (b) PPLA 400, (c) PPLA2000.

Fig 4(A)–4(C) shows the SEM micrographs of some representative samples. As predicted, the typical brittle fracture morphology of the pure PLA can be observed in Fig 4(A). A comparison of the tensile cross-section micrographs of the PLA blends plasticized with 10 wt% of PEG and the pure PLA shows that the dispersed PEG phase and dimples were observed where the PEG was identified as particles in the PLA matrix. With an increasing *M*_w_, the particles and matrix were closely combined and thereby played a role in the toughening effect. This occurred because stress transfer from the PLA matrix to toughing particles when the materials suffered an external force, which in turns to improve the ductility of PLA [25–27]. However, the poor interfacial bonding and uneven dispersion led to a limited increase in the ductility of the materials, which was consistent with the mechanical test results.

### Thermal properties of PLA blends

**Fig 5:**
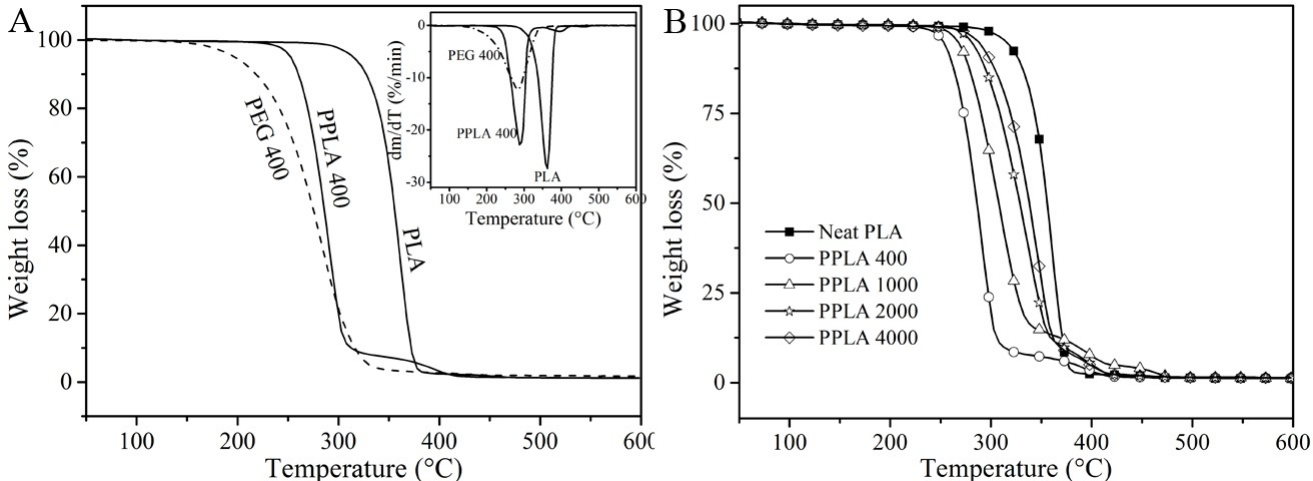
Thermogravimetric curves of: (A) TGA-DTG curves for pure PLA, PEG 400 and PPLA 400. (B) pure and plasticized PLA.

In previous studies of plasticized modification, it was often neglected to take into account the effect of plasticizers on the thermal stability of the polymer matrix. For this reason, TGA measurements were conducted to analyze the thermal properties of the PLA blends. Fig 5 shows that the addition of plasticizers had a great influence on the thermal degradation behavior of the PLA matrix and the decomposition temperatures all shifted systematically to lower temperatures compared to the pure PLA. The differential thermogravimetric (DTG) curves in Fig 5A indicate that the thermal decomposition mechanism of the PLA matrix changed in the presence of the plasticizers. The shift was evidently more obvious when the *M*_w_ of the PEG was lower as shown in Fig 5B. For example, it was observed that the decomposition onset temperature (*T*_o_) and the temperature at maximum degradation rate (*T*_max_) of the blend PLA/PEG 400 shifted from 338 to 264°C and from 360 to 291°C, respectively. This was due to the PEG degradation when the initial degradation temperature reached 235°C and promoted PLA degradation [28]. It is evident that the smaller the M_w_ of the PEG, the more likely it decomposes.

**Fig 6:**
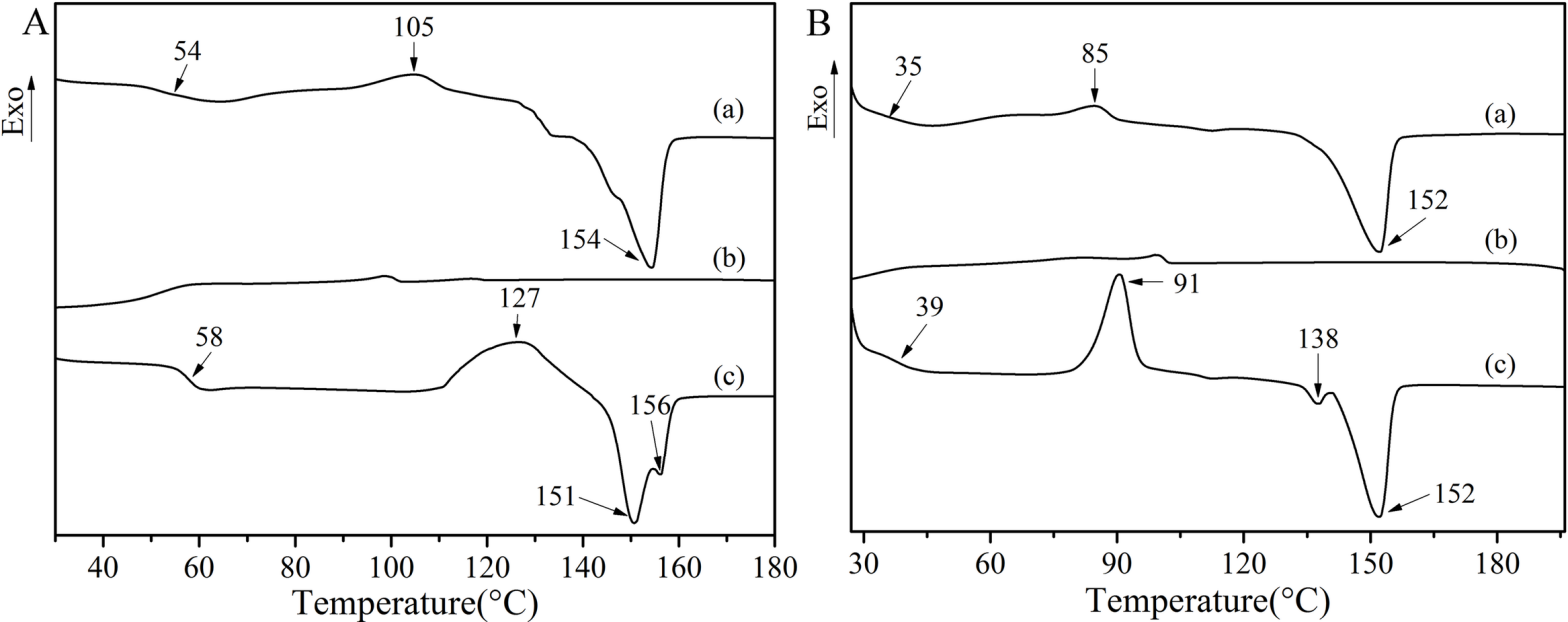
DSC curves of: A. pure PLA; B. PPLA 400: (a) the first heating curve, (b) cooling curve, and (c) the second heating curve.

To elucidate the roles of the PEGs in the plasticizing of the PLA, blends of PLA containing 10 wt% of PEGs were prepared. Here, non-isothermal studies by DSC make it possible to trace the changes in the plasticity, the nucleation, and the crystallinity based on *T*_g_, *T*_c_, and *χ*_c_ respectively. Fig 6 shows a complete cycle of DSC thermo-grams of native PLA and PLA mixed at 10 wt% with PEG 400. For pure PLA, the *T*_g_ at 54°C followed by *T*_c_ at 105°C is identified prior to *T*_m_ at 154°C at the first heating (Fig 6A(a)), while no melt crystallization is observed upon cooling (Fig 6A(b)). Compared with the thermogram of the first heating, the cold crystallization peak of the second heating shifted to a higher temperature, indicating that the chain-packing requires more thermal energy [29]. Furthermore, the melting peak changed from a single peak to double peaks, in which the lower temperature peak can be understood as the crystal melting peak of the PLA and the higher temperature peak as the melting peak of the original crystalline grain recrystallization.

For PPLA 400 (Fig 6B), the thermogram of the blend is quite similar to that of the native PLA. As compared with the second heating (Fig 6A(c) and 6B(c)), which refers to a deletion of the thermal history, the scans provided important information, namely (i) a significant reduction of *T*_g_ of the PLA from 58 to 39°C was observed, (ii) a cold-crystallization temperature reached as low as 91°C, and (iii) two melting temperatures that are lowered to 138°C and 152°C. The lowering of *T*_g_ and *T*_c_ implies that an increase in chain mobility and that chain-packing occurred easily [30]. In other words, the crystallization can start at a lower temperature than for the native PLA upon heating. The great decrease in *T*_g_ of the blend might be related to a probable structural change of the PLA matrix. Furthermore, the melting peak of the plasticized blends changed to a lower temperature due to the amorphous structure of the PEG. Compared with the pure PLA, the lower temperature melting peak was weakened and the higher temperature melting peak was enhanced, which suggested that the crystal structure of the PLA was changed in the presence of PEG [31].

**Fig 7:**
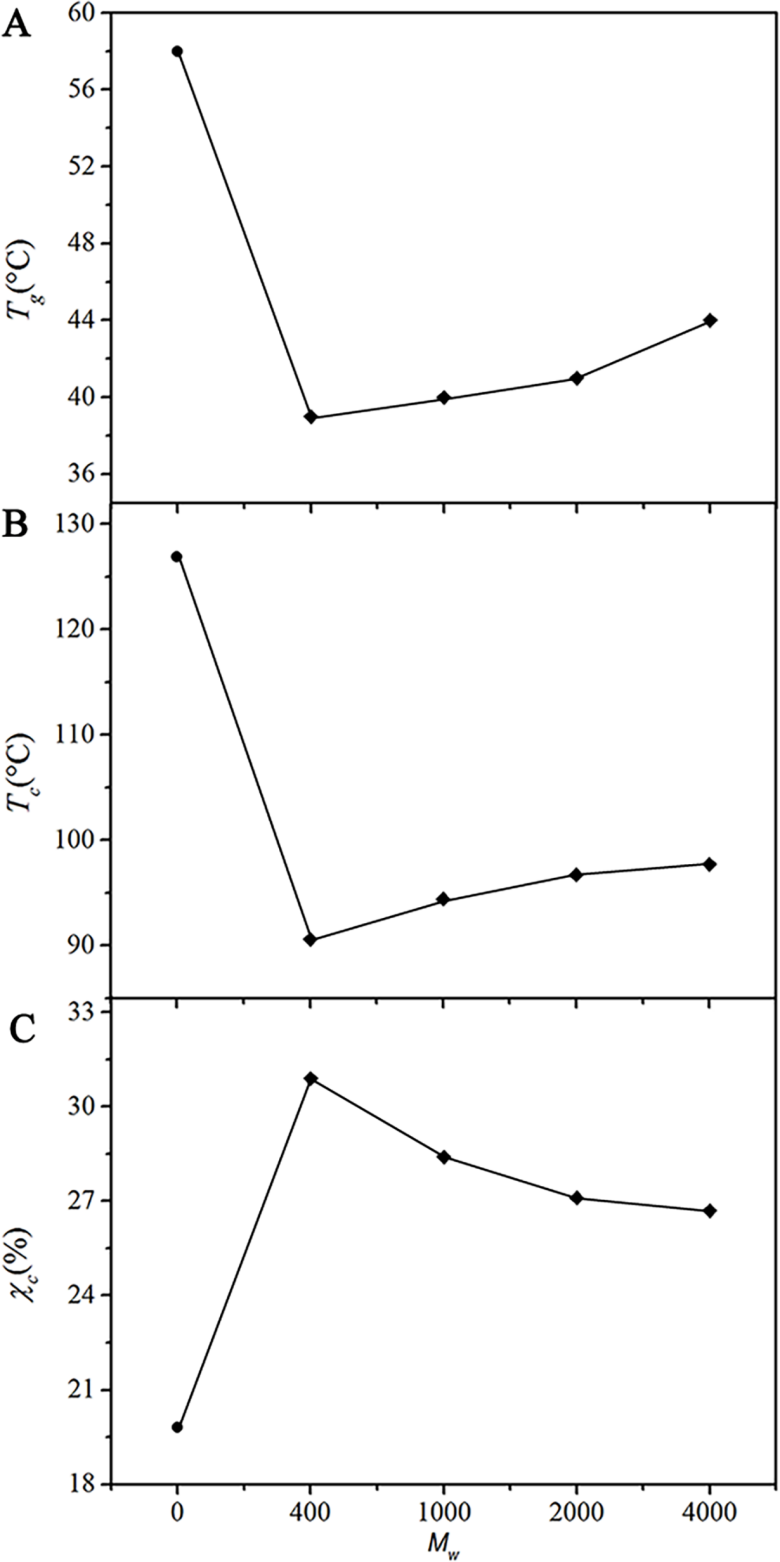
(A) *T*_g_. (B) *T*_c_ and (C) *χ*_c_ of PLA blended with 10 wt% PEG at various *M*_w_s (from the second heating curve): (●) neat PLA, (◆) PPLA.

Fig 7A shows the glass transition temperatures (*T*_g_s) of the PPLA mixed with a variety of PEGs. It was clear that the lower the *M*_w_ of the PEG, the more significant the *T*_g_ reduction was. For example, PPLA 400 and PPLA 1000 show *T*_g_s as low as 39 and 40°C, whereas PPLA 2000 and PPLA 4000 exhibit higher *T*_g_s at 41 and 44°C, respectively. Here, the changes in *T*_c_ and *χ*_c_ were also observed (Fig 7B and 7C). The increase in T_c_ along with the larger *M*_w_ reflected the slower crystallization rate of the PLA [30]. Namely, the large *M*_w_ of PEG might disturb the movement of PLA chain to form nucleation. In addition, the *χ*_c_ of the blends decreased with increasing *M*_w_, which indicated the decrease in the chain-packing.

### Estimation of surface free energy of PLA blends

According to the Zisman critical free energy theory [32], the interfacial compatibility of the composites is related to the free energy of the two phases. When the surface free energy of the PLA is equal to or lower than the fiber, it can form a stable fusion. The greater the difference, the better the interface obtained. CA analysis was performed to investigate the surface free energy and its dispersive, polar component of the PLA materials; the results are shown in Table 2.

**Table 2:** Results of surface tension analysis.

Sample	Contact angle (°)			γ_s_^D^	γ_s_^P^	γ_s_
	Water	Ethylene glycol	Atolin			
PLA	76.87±0.76	68.25±0.21	57.40±0.83	30.52	7.30	37.82
PPLA 400	74.38±3.22	74.20±0.00	61.95±1.33	13.58	23.49	37.07
PPLA 1000	73.85±0.00	70.53±1.02	64.18±1.17	9.57	9.32	18.89
PPLA 2000	64.55±0.78	77.00±0.00	61.10±3.18	4.75	8.22	12.97
PPLA 4000	57.93±7.20	74.25±0.85	52.27±2.99	3.14	25.54	28.68

Referring to the Owens-Wendt [17] plotting procedure, the polar and dispersive components of the polymer surface free energy, γ_s_^P^ and γ_s_^D^, were assessed respectively. The equation (Eq 2) is a linear equation, y = mx+ b, while the slope equals (γ_D_^P^)^1/2^ and the Y-axis intercept is (γ_S_^D^)^1/2^. As a result, the surface free energy and its components in the plasticized PLA blends are listed in Table 2. As is evident, the surface free energy of native PLA was low and consisted mainly of the dispersive component, which reflects the poor polarity of the untreated PLA materials. By analyzing the components of surface free energy, it was found that the polar component increased markedly after plasticization, while the surface free energy decreased in different degrees. These results show that plasticization is important in improving the polarity of the PLA materials and is conducive to the formation of a good interfacial compatibility between the PLA and the polar fibers.

### Interfacial morphologies of PLA/WF composites

**Fig 8:**
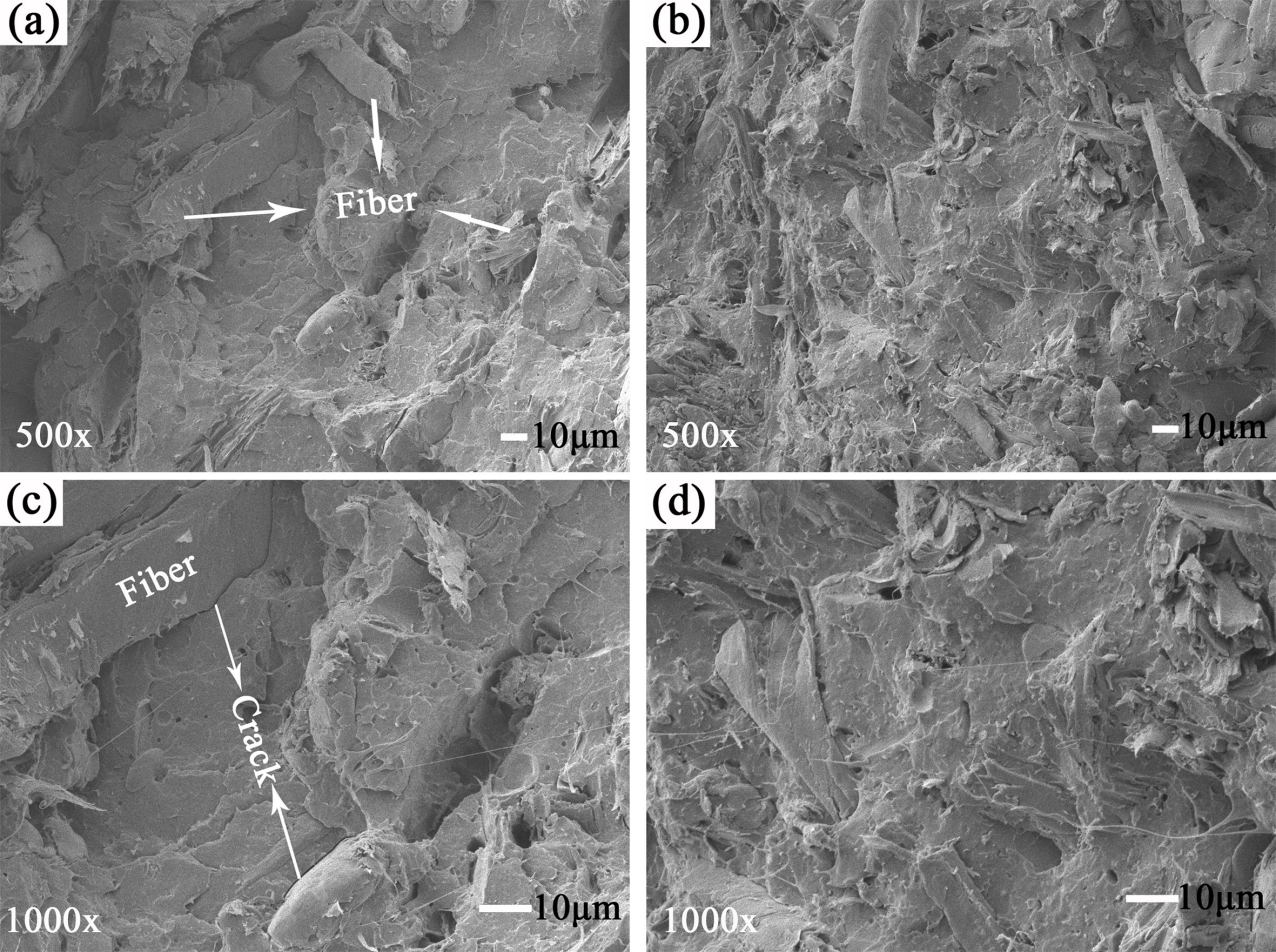
SEM micrographs of the PLA/WF composite: (a) and (c) PLA; (b) and (d) PPLA 2000.

Many properties of composite can be affected by morphology, which was analyzed by SEM in the experiments. Fig 8 shows the SEM micrographs of the composites prepared with pure PLA and PPLA as matrix resin. In Fig 8(A) and 8(C), the cross-section of the PLA/WF composite was rough. The exposed fiber and cracks between the WF and the PLA in the untreated composite are easily observed. The findings suggested that the interaction between the filler and the matrix was poor, resulting in a decreased interfacial compatibility.

The plasticized composites in the micrographs (Fig 8(B) and 8(D)) show that the WF was coated well with the PPLA resin, demonstrating a compatible structure between the PPLA and the WF. The enhancement of the interfacial compatibility was attributed to the increased flexibility and reduced free energy of the matrix resin. The results supported the enhancement of the comprehensive properties by the addition of the PEG plasticizer, which is analyzed below.

### Mechanical properties of PLA/WF composites

**Fig 9:**
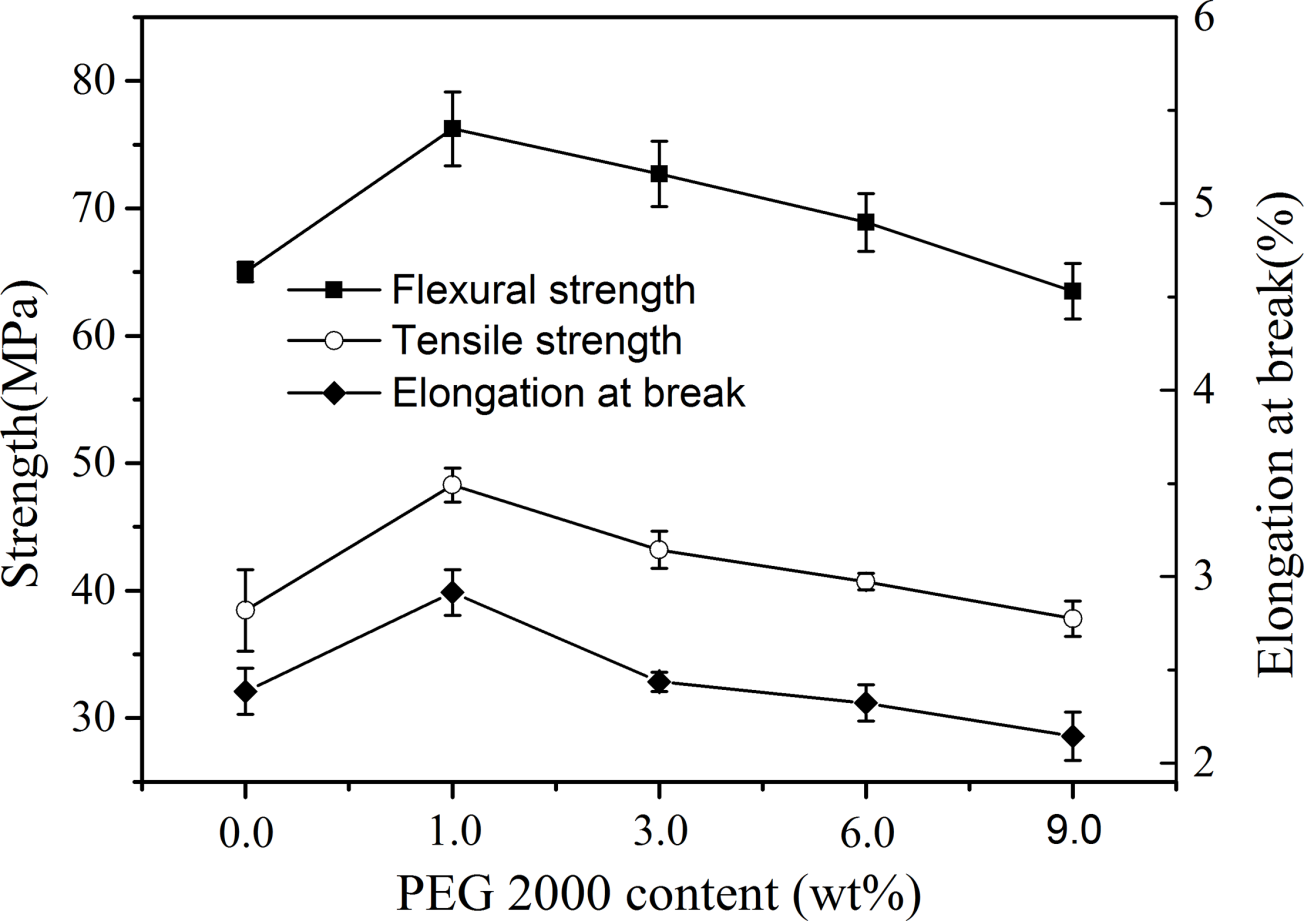
Mechanical strength of PLA/WF composites with different amounts of PEG2000.

The elongation at break and the tensile and bending strength measurements were carried out to evaluate the mechanical properties of the composites. In Fig 9, the relationship between the strength and the PEG content in the composites is demonstrated. Clearly, for the plasticized specimens, all the PPLA/WF composites had higher flexural and tensile strengths than the untreated composites. The more the PEG content, the smaller the difference was obtained between them because the tensile strength of the PPLA matrix decreased more rapidly with the plasticizer content than that of the interfacial bonding strength. The flexural strength of the samples exhibited a similar difference. It was noted that the tensile and flexural strengths both displayed a maximum value at a PEG amount of 1.0 wt%. When 1.0 wt% PEG was added, a considerable increase in the elongation at break occurred. All the experimental results indicated that PEG played an important role in improving the properties of the composites because the plasticized PPLA increased the ductility of the matrix and reduced its surface free energy, which was helpful in improving the coating effect of the matrix resin on the WF filler and enhancing the interfacial bonding strength.

### Melting behavior of PLA/WF composites based on the DSC

**Fig 10:**
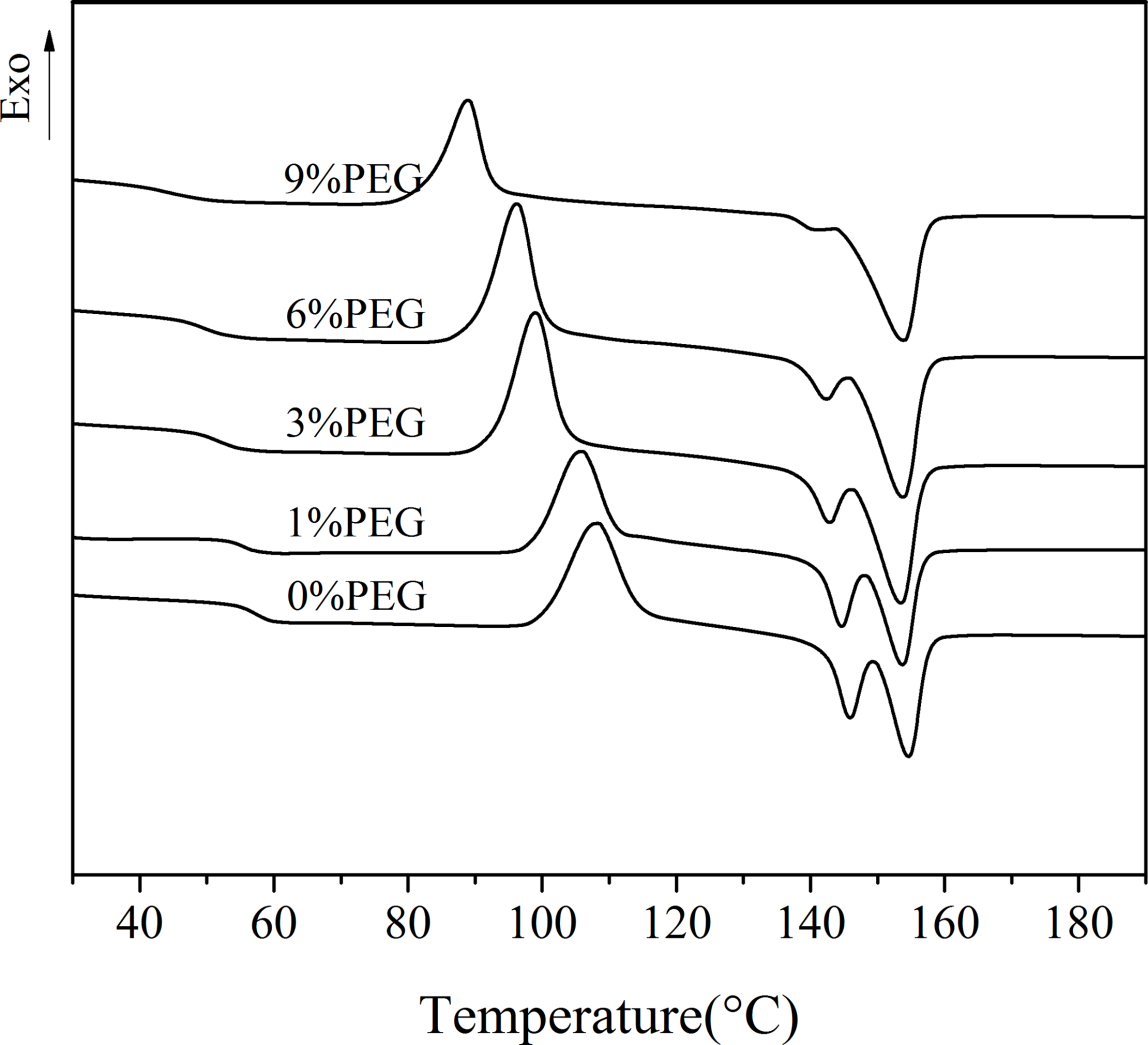
DSC traces (the second heating) of the melting behavior of the composites with different amounts of PEG2000.

Fig 10 shows the melting behavior of the composites at plasticizer contents ranging from 1 to 9 wt%. Significant decreases in *T*_g_, *T*_c_, and *T*_m_ of the plasticized WF/PLA composites were observed. Furthermore, all samples showed double melting endotherms for crystallized nonisothermally from the melt. The area of the low melting endotherm decreased and the area of the high melting endotherm increased with the increase in the PEG content. The results showed that the addition of PEG enhanced the movement ability of the PLA segments and played a plasticizing role in the WF/PLA composites. The plasticizing effect was gradually weakened, which was consistent with the trend of elongation at break of the plasticized composites.

### Thermal stability of PLA/WF composites TGA

**Fig 11:**
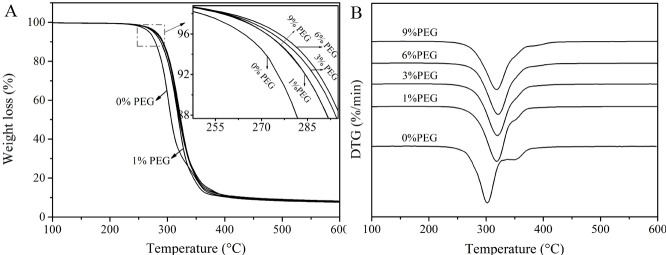
Thermogravimetric curves of the composites with different contents of PEG 2000.

As the content of the PEG varied, the compatibility of the composite varied accordingly, the thermal decomposition behavior of the composite was affected. Therefore, it was necessary to analyze the thermal stability of the composite; the results are shown in Fig 11.

As shown in Fig 11, the thermal degradation temperatures of the composites with the plasticized PLA blends all increased and gradually moved towards a higher temperature with the increasing PEG content. Meanwhile, the small cromion of the DTG curves weakened gradually until it disappeared compared with the un-plasticized PLA based composite. This suggested that the plasticized modification brought about an improvement in compatibility and thermal stability of the WF/PLA composites.

## Conclusions

A series of injection-molded PPLA were prepared by melt blending. FTIR-ATR indicates that the -CH_2_- chain segment of the PEG is introduced into the PLA matrix and that the hydrogen bonding interaction increases. DSC performed on the blends containing an increasing *M*_w_ of PEG confirms this hypothesis since all PPLAs show drastic decreases in both *T*_g_ and *T*_c_, which supports the statement that plasticization has succeeded. Further, using PPLA 400 as an example, a high *χ*_c_ (from 20% to 41%) and significant decreases in *T*_g_ (from 58 to 39°C) and *T*_c_ (from 127 to 91°C) were found. It is clear that the existence of the plasticizer breaks the balance of amorphous and crystalline domains in the PLA and improves the ductility of the blends, which is also confirmed by the results of the SEM and tensile test. In addition, the use of plasticizers can play a role in reducing the interfacial energy of PLA, leading to an increase in polarity and a decrease in surface free energy. All the results suggest that PPLA as the matrix resin of the composites improves its compatibility with a polar filler (WF) and then enhances the comprehensive properties of the composites. This is consistent with the test results of the PPLA/WF composites, which mainly focus on mechanical properties and thermal stability.

## Supporting information

S1 Data Raw data of this work. (ZIP)
